# Beyond Geography: Using a Cluster‐Based Analysis to Conceptualize the Rurality of Veterans Health Administration Hospitals

**DOI:** 10.1111/jrh.70195

**Published:** 2026-08-10

**Authors:** Alexandra B. Caloudas, Michihiko Goto, M. Bryant Howren, Zachary Burningham, Diana J. Govier, Travis I. Lovejoy, Eric R. Litt, James W. Watts, Sarah S. Ono, Kristen Wing, Peter J. Kaboli

**Affiliations:** ^1^ Department of Veterans Affairs (VA) Veterans Health Administration (VHA), Office of Rural Health (ORH), Veterans Rural Health Resource Center—Salt Lake City (VRHRC‐SLC), VA Salt Lake City Healthcare System Salt Lake City Utah USA; ^2^ VHA Health Systems Research, Center for Innovations in Quality Effectiveness, and Safety, Michael E. DeBakey VA Medical Center Houston Texas USA; ^3^ South Central VA Health Care Network, South Central Mental Illness Research, Education and Clinical Center, Michael E. DeBakey VA Medical Center Houston Texas USA; ^4^ Menninger Department of Psychiatry and Behavioral Sciences Baylor College of Medicine, One Baylor Plaza Houston Texas USA; ^5^ VHA Office of Rural Health, Veterans Rural Health Resource Center—Iowa City (VRHRC‐IC), Iowa City VA Health Care System Iowa City Iowa USA; ^6^ VHA Health Systems Research, Center for Access & Delivery Research and Evaluation, VA Iowa City Healthcare System Iowa City Iowa USA; ^7^ Department of Internal Medicine University of Iowa, Carver College of Medicine Iowa City Iowa USA; ^8^ VHA Health Systems Research, Informatics, Decision‐Enhancement and Analytic Sciences Center, Salt Lake City Veterans Affairs Medical Center Salt Lake City Utah USA; ^9^ Department of Internal Medicine, Division of Epidemiology University of Utah Salt Lake City Utah USA; ^10^ VHA Office of Rural Health, Veterans Rural Health Resource Center—Portland, VA Portland Health Care System Portland Oregon USA; ^11^ VHA Health Systems Research, Center to Improve Veteran Involvement in Care, VA Portland Health Care System Portland Oregon USA; ^12^ Department of Psychiatry School of Medicine Oregon Health & Science University Portland Oregon USA; ^13^ School of Public Health Oregon Health & Science University–Portland State University Portland Oregon USA; ^14^ North Florida–South Georgia Veterans Health Care System Gainesville Florida USA; ^15^ VHA Office of Rural Health Washington District of Columbia USA

**Keywords:** hospitals, rural, veterans

## Abstract

**Purpose:**

Rural veterans experience challenges and strengths related to military service and rural residence. Defining rurality for healthcare facilities is vital for allocating resources, estimating impacts of rural health initiatives, and evaluating rural veteran access and outcomes. While geolocation is one way of conceptualizing rural healthcare facilities, rurality exists on a continuum and is influenced by many factors. The purpose of this work was to create an empirically informed taxonomy of VHA acute care hospitals that moves beyond a geography‐based classification alone and to propose facility‐level variables that can be used to measure rural veterans’ access to hospital‐based care.

**Methods:**

We conducted a cluster analysis of 110 Veterans Health Administration (VHA) acute care hospitals using k‐means clustering. Hospital‐level variables included number of enrollees, number and percentage of rural and highly rural enrollees, VHA hospital complexity classification level (low, medium, high), and mean drive times for veterans to access VHA care (primary, secondary, tertiary).

**Findings:**

Three clusters emerged. The Low Rural Serving (LRS) Cluster (*N* = 40) comprised 39 high complexity hospitals, with an average of 16.5% of veteran enrollees residing in rural areas. The Medium Rural Serving (MRS) Cluster (*N* = 26) contained all high complexity hospitals, with an average of 32.8% of veteran enrollees residing in rural areas. The High Rural Serving (HRS) Cluster (*N* = 44) comprised 10 low, 12 medium, and 22 high complexity hospitals, with an average of 61.9% of veteran enrollees residing in rural areas. The average percentage of highly rural veteran enrollees was 1.0% for LRS, 1.6% for MRS, and 9.7% for HRS. Average drive times to primary, secondary, and tertiary care increased incrementally across clusters.

**Conclusions:**

Using 9 hospital‐level factors that may characterize degree of hospital rurality, we identified 3 clusters that differentiate VHA hospitals and the rural populations they serve.

## 1 Background

Rural Americans have a rich history of US military participation and volunteer for military service at higher rates than their urban counterparts [1]. This heterogeneous population experiences both challenges and strengths related to the intersection of military service and rural residence [2]. Military participation elevates risk for many physical and mental health conditions (e.g., musculoskeletal disorders, post‐traumatic stress disorder), and rural residence is associated with higher rates of chronic conditions (e.g., heart disease, stroke) and healthcare access barriers [3, 4, 5, 6]. While only 20% of US residents live in rural areas, rural veterans constitute almost 33% of the 9 million veterans enrolled in the Veterans Health Administration (VHA), the nation's largest integrated healthcare system [7, 8, 9].

Defining “rural” is complex given that rural areas are characterized by immense heterogeneity in landscapes, local norms and values, and economic structures [10]. While rurality can be associated with characteristics like geographic isolation and low population density, it is not solely defined by these factors [11]. Yet, the most frequently used definitions of rurality, based on US Census Bureau's and Office of Management and Budget's classification systems, are geographic‐based definitions that label rural areas as those that are not urban or metropolitan [12, 13]. No uniform definition of rurality has been adopted across the federal government [12], and attempts to define rurality are driven by specific intentions of the users [11]. As a result, there are meaningful differences in who is characterized as rural when comparing common definitions of rurality [14]. These differences matter because how rurality is defined has clear implications for who is eligible for rural‐focused programs designed to enhance access to healthcare [14]. Further, relying on geography alone to characterize rurality, particularly when using rural–urban dichotomies, may mask other underlying factors that have important implications for healthcare access like the availability of local healthcare providers and access to healthcare resources in surrounding areas [15, 16].

Within VHA, the definition of rurality informs both how healthcare is delivered and how funds are allocated to support advancements in care. The VHA Office of Rural Health (ORH), whose mission is to “honor America's rural veterans by providing exceptional healthcare that improves their health and well‐being,” funds research, quality improvement, and other programs or initiatives that strengthen rural veterans’ health and expand their healthcare access [9]. A critical part of allocating ORH funds involves determining which initiatives have the greatest impact, a process that requires a clear definition of rurality for both individuals and locations of care.

Since 2014, VHA has used a geography‐based definition to characterize the rurality of veterans and facilities; this definition is based on the Rural–Urban Commuting Areas (RUCA) system developed by the Department of Agriculture Economic Research Service and the Health Resources and Services Administration Office of Rural Health Policy [17, 18]. Although VHA has applied this definition to both veterans and facilities (including medical centers and primary care clinics), the focus of the current work is on conceptualizing the rurality of VHA hospitals in light of the populations they serve. While prior work has focused on rural veterans’ access to VHA facilities [19], less attention has been paid to how VHA's geography‐based definition, as applied to VHA facilities, may conceal important nuances in how healthcare facilities function, how care is delivered, and rural veterans’ actual patterns of healthcare use. For example, consider a low resourced rural acute care hospital that serves a large number of rural veterans where the nearest high‐resourced hospital is 200 miles away. This facility likely has distinct needs around staffing, care coordination, and transporting patients with urgent needs than a rural facility that is located within 30 min of a high complexity urban hospital. A nuanced approach in supporting each facility, one that recognizes rurality as a continuum, is thus warranted.

Having access to hospitals providing acute care services is especially important for rural veterans given that these hospitals often provide care for urgent and life‐threatening conditions [20]. Geography‐based definitions of rurality may not, however, account for non‐geographic factors that shape how acute care hospitals function—such as the services they offer, the populations they serve, or the barriers facing those in need of their services. While additional research is needed to identify the full range of relevant non‐geographic factors, hospital complexity, number of rural veteran enrollees, and mean drive times for veterans to VHA primary, secondary, and tertiary care may be valuable metrics for understanding rural veterans’ access to acute care. Hospital complexity reflects the types and acuity of care offered by hospitals, with higher complexity being associated with greater access to specialized services as well as important healthcare outcomes [21]. Similarly, the number of rural veterans enrolled in a VHA hospital may serve as an indicator of both how the hospital functions and the degree to which it operates as a primary healthcare resource for rural veterans who face heightened healthcare needs and access barriers. Finally, mean drive times reflect the burden in accessing care and have also been linked with patient outcomes [22].

The primary objective of this work was to use a cluster‐based analysis to create a VHA facility‐level taxonomy, focused on acute care hospitals, to provide a more empirically informed definition of rural locations of care beyond a geography‐based classification alone. A secondary objective was to propose facility‐level factors that can be used to measure and better understand access to hospital‐based care for rural veterans.

## 2 Methods

### 2.1 Data and Participants

#### 2.1.1 VHA Hospital Data

VHA hospital data were obtained from the Veteran Affairs Site Tracking (VAST) system database [23]. The VAST system maintains an inventory and rurality profile of all VHA service sites using the RUCA classification system [17, 18, 23]. VAST monitors all VHA service sites, their locations, types of services provided, and geocoded attributes (e.g., longitude, latitude, rurality). The latitude and longitude of each facility are mapped to the RUCA codes. We limited analyses to 110 VHA hospitals that provide acute care services (i.e., inpatient medical/surgical and emergency/urgent care), given the important function that acute care hospitals serve in addressing rural veterans’ urgent and emergent health concerns [20]. VHA hospital selection was guided by a list of acute care hospitals obtained from VHA's Hospital Medicine Analytics Team, which maintains data on VHA hospitals. Fiscal year (FY) 2023 data were used.

#### 2.1.2 Veteran Enrollee Data

The Planning Systems Support Group (PSSG) Geocoded Enrollee File contains residential information and geographic attributes (e.g., census tract, longitude, latitude, rurality) of all veteran enrollees [24]. PSSG organizes data into analytic datasets by FY and quarter, identifying each veteran's nearest VHA sites of care (i.e., primary, secondary, and tertiary) and calculating estimated drive time and distance from the enrollee's geocoded address. FY 2023 data were used.

### 2.2 Variables

#### 2.2.1 Rurality

VHA's area‐based definition of rurality was used to define rurality as it applies to veterans and VHA hospitals. Veterans and VHA facilities geographically located in RUCA 1.0 or 1.1 census tracts are designated as urban by VHA, while veterans and facilities located in RUCA 10.0 census tracts are designated as highly rural. All remaining RUCA code census tracts are designated as rural. Veterans living on insular islands are not coded using RUCA but are designated as “insular island” by VHA. For our purposes, those defined as rural, highly rural, or insular islands using VHA criteria were broadly labeled as “rural.” Veterans or hospitals labeled as “highly rural” were those which met VHA's definition of highly rural. While some patients met the definition of highly rural, no VHA hospitals in the current work were highly rural or on an insular island.

#### 2.2.2 Hospital Complexity

VHA uses 5 facility complexity levels to categorize VHA facilities including 1a, 1b, 1c, 2, and 3, with 3 being the least complex and 1a being the most complex. These five distinct complexity levels were maintained for analyses, but levels 1a, 1b, and 1c were collapsed and facility complexity was simplified to level 1 (high), level 2 (medium), and level 3 (low) complexity when reporting cluster analysis results.

#### 2.2.3 Veteran Enrollees

Veteran enrollees were defined as veterans who enrolled in VHA, regardless of their use of VHA services. Enrollees were further subdivided into urban, rural, highly rural, and insular island based on their addresses and according to VHA's area‐based definition of rurality. Veteran enrollees were assigned to a given VHA hospital by PSSG based on geographical proximity (i.e., those living closest to a hospital were considered an enrollee of that hospital).

#### 2.2.4 Drive Times

Mean drive times for VHA enrollees to reach their nearest VHA facilities that provide primary, secondary, and tertiary care are calculated by the Geospatial Services Support Center (GSSC) based on traffic patterns on Wednesdays at 10am. Calculations are reported in minutes and integrate latitudes and longitudes of veteran addresses, travel time, and road conditions to estimate average drive times for veterans to access VHA care [25].

### 2.3 Data Analyses

This work describes VHA hospitals using several indicators to characterize their rurality and the extent to which they serve rural populations. We first identified the total number of VHA acute care hospitals geolocated in urban and rural locations based on the VHA's definition (*N* = 110). We next extracted data on each hospital across nine variables including hospital complexity (low, medium, high), total number of veteran enrollees, number of rural veteran enrollees, percent of rural veteran enrollees, number of highly rural veteran enrollees, percent of highly rural veteran enrollees, mean drive times for enrollees to reach VHA primary care, mean drive times for enrollees to reach VHA secondary care, and mean drive times for enrollees to reach VHA tertiary care.

We then employed k‐means clustering [26] to evaluate whether the VHA hospitals formed distinct groups based on facility size, rural veteran enrollment, geographic access to care, and facility complexity. K‐means is a machine learning algorithm that partitions facilities into clusters by minimizing within‐cluster variation (within‐cluster sum of squares) using squared Euclidean distances. The nine facility‐level variables were considered when clustering facilities. Because the distributions of the number of highly rural enrollees and the percent highly rural were strongly right‐skewed, we applied log(*x* + 1) transformations to these two measures variables prior to conducting cluster analyses.

To accommodate the mix of ordinal (facility complexity) and continuous variables and to reduce dimensionality while accounting for scale differences and collinearity, we first performed Factor Analysis of Mixed Data (FAMD) and used the resulting component scores as inputs for clustering [27, 28]. The number of retained components was selected based on the scree plot (retaining components explaining the majority of total inertia), after evaluating up to eight components. K‐means models were estimated for *k* = 2 through *k* = 5 clusters with 50 random initializations to improve solution stability. The optimal number of clusters was selected by comparing average silhouette widths across candidate solutions (computed using Euclidean distances in the retained FAMD component space) while considering interpretability of the resulting groups. An additional investigation of other cluster options ranging from *k* = 2 to *k* = 5 (i.e., sensitivity analyses) validated the selection of *k* = 3 as the ideal and most interpretable solution. All analyses were performed using R version 4.5.2 (R Foundation for Statistical Computing, Vienna, Austria) and FactoMineR package [27, 28].

Once clusters were identified, we calculated unweighted descriptive statistics for the variables and then characterized similarities and differences between clusters. Several hospital‐level variables used in the cluster analysis were themselves averages (e.g., mean number of enrollees per hospital, mean drive times). Therefore, subsequent descriptive analyses to characterize clusters involved calculating the mean of these hospital‐level means. In other words, we calculated the mean average of each hospital's mean value, such as the mean of hospital‐level mean drive times or enrollee counts. Institutional Review Board evaluation was not required for acquisition and analyses of these data given the work was conducted as a non‐research operational analysis for the VHA Office of Rural Health. Access to the data was granted by the National Data Systems group under the VHA Office of Health Informatics Health Information Governance.

## 3 Results

**FIGURE 1:**
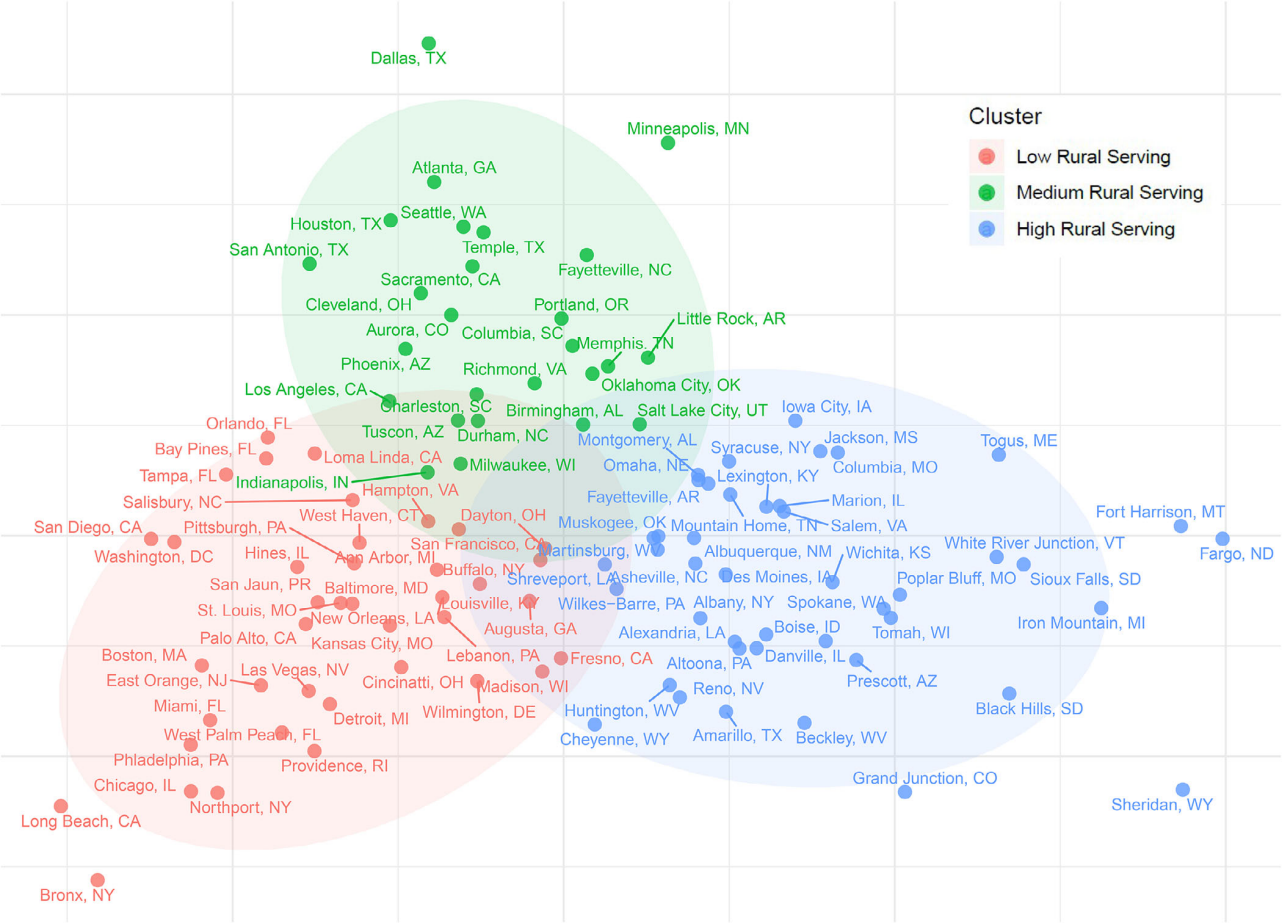
Cluster analysis of 110 VHA hospitals. *Note*: This figure depicts clustering in 2‐dimensional space. However, the analysis used 5 dimensions, and facilities in this figure may appear outside the cluster ellipses in this 2‐dimensional depiction.

Of the 110 hospitals included in the cluster analysis, three distinct clusters were identified (Table 1 and Figure 1). See Table 2 for a complete list of hospitals by cluster and location. Data are presented on the number and percentage of rural veteran enrollees, which is inclusive of highly rural veteran enrollees. Number and percentage of highly rural veteran enrollees are also reported separately to highlight this clinically and programmatically distinct veteran group.

**TABLE 1:** Characteristics of VHA hospital by cluster (*n* = 110).

Cluster	VHA hospital complexity	Mean (range) number enrollees	Mean (range) number of rural enrollees	Mean (range) percent rural^a^	Mean number (range) of highly rural enrollees	Mean percent (range) highly rural	Mean drive time to (range) primary care in minutes	Mean drive time (range) to secondary care in minutes	Mean drive time to (range) tertiary care in minutes
1 Low Rural Serving 36% *N* = 40	2% medium complexity (*n* = 1/40) 98% high complexity (*n* = 39/40)	66,911 (21,992–146,374)	9965 (8– 24,231)	16.5% (0.04%‐ 47.6%)	580 (0–3071)	1.0% (0–6.7%)	17.4 (10.6– 28.5)	34.9 (14.7– 57.2)	63.7 (15.0– 208.8)
2 Medium Rural Serving 24% *N* = 26	100% high complexity (*n* = 26)	112,215 (63,373–200,392)	35,016 (16,445– 61,896)	32.8% (15.0%‐56.1%)	2741 (491– 9919)	2.6% (0.5%‐ 8.1%)	21.5 (15.6–30.5)	49.1 (25.1– 75.1)	68.9 (29.9–130.1)
3 High Rural Serving 40% *N* = 44	23% low complexity (*n* = 10/44) 27% medium complexity (*n* = 12/44) 50% high complexity (*n* = 22/44)	40,270 (9,954– 71,792)	24,157 (7,644– 40,364)	61.9% (33.8–100)	3615 (839– 9620)	9.7% (2.6–28.6)	29.3 (21.7– 58.5)	62.4 (32.1– 130.8)	166 (58.7– 427.7)

^a^ Includes rural, highly rural, and insular island enrollees.

**TABLE 2:** List of Veterans Health Administration hospitals by cluster and location.

Cluster 1: Low Rural Serving	Cluster 2: Medium Rural Serving	Cluster 3: High Rural Serving
Ann Arbor, MI	Atlanta, GA	Albany, NY
Augusta, GA	Aurora, CO	Albuquerque, NM
Baltimore, MD	Birmingham, AL	Alexandria, LA
Bay Pines, FL	Charleston, SC	Altoona, PA
Boston, MA	Cleveland, OH	Amarillo, TX
Bronx, NY	Columbia, SC	Asheville, NC
Buffalo, NY	Dallas, TX	Beckley, WV
Chicago, IL	Durham, NC	Black Hills, SD
Cincinnati, OH	Fayetteville, NC	Boise, ID
Dayton, OH	Houston, TX	Cheyenne, WY
Detroit, MI	Indianapolis, IN	Clarksburg, WV
East Orange, NJ	Little Rock, AR	Columbia, MO
Fresno, CA	Los Angeles, CA	Danville, IL
Hampton, VA	Memphis, TN	Des Moines, IA
Hines, IL	Milwaukee, WI	Fargo, ND
Kansas City, MO	Minneapolis, MN	Fayetteville, AR
Las Vegas, NV	Oklahoma City, OK	Fort Harrison, MT
Lebanon, PA	Phoenix, AZ	Grand Junction, CO
Loma Linda, CA	Portland, OR	Huntington, WV
Long Beach, CA	Richmond, VA	Iowa City, IA
Louisville, KY	Sacramento, CA	Iron Mountain, MI
Madison, WI	Salt Lake City, UT	Jackson, MS
Miami, FL	San Antonio, TX	Lexington, KY
New Orleans, LA	Seattle, WA	Marion, IL
Northport, NY	Temple, TX	Marion, IN
Orlando, FL	Tucson, AZ	Martinsburg, WV
Palo Alto, CA		Montgomery, AL
Philadelphia, PA		Mountain Home, TN
Pittsburgh, PA		Muskogee, OK
Providence, RI		Omaha, NE
Salisbury, NC		Poplar Bluff, MO
San Diego, CA		Prescott, AZ
San Francisco, CA		Reno, NV
San Juan, PR		Salem, VA
St. Louis, MO		Sheridan, WY
Tampa, FL		Shreveport, LA
Washington, DC		Sioux Falls, SD
West Haven, CT		Spokane, WA
West Palm Beach, FL		Syracuse, NY
Wilmington, DE		Togus, ME
		Tomah, WI
		White River Junction, VT
		Wichita, KS
		Wilkes‐Barre, PA

**Cluster 1: Low Rural Serving**: Hospitals (*n* = 40) falling into cluster 1 were 98% high complexity and 2% medium complexity. The mean of hospital‐level means indicated that 16.5% of enrollees were rural (including rural, highly rural, or insular island) veterans, with 1% meeting VHA's definition of highly rural. The mean of means for veteran drive times to care was shorter than in other clusters: 17.4 min to primary care, 34.9 min to secondary care, and 63.7 min to tertiary care. The mean of hospital‐level means for enrollees at these 40 Low Rural Serving hospitals was 66,911 (range: 21,992–146,374).

**Cluster 2: Medium Rural Serving**: Hospitals (*n* = 26) in cluster 2 were all high complexity hospitals. The mean of hospital‐level means indicated that 32.8% of enrollees in this cluster were rural (including rural, highly rural, or insular island) veterans, with 2.6% meeting VHA's definition of highly rural. The mean of means for veteran drive times to care was intermediate compared to other clusters: 21.5 min to primary care, 49.1 min to secondary care, and 68.9 min to tertiary care. The mean of hospital‐level means for enrollees at these 26 Medium Rural Serving hospitals was 112,215 (range 63,373–200,392).

**Cluster 3: High Rural Serving**: Cluster 3 (*n* = 44) was composed of a mixture of low (23%), medium (27%), and high complexity (50%) hospitals. The mean of hospital‐level means indicated that this cluster had the highest percentage of rural veteran enrollees among all three clusters, with 61.9% classified as rural (rural, highly rural, or insular island) and 9.7% meeting VHA's definition of highly rural. The mean of means for veteran drive times to care was longest compared to other clusters: 29.3 min to primary care, 62.4 min to secondary care, and 166.0 min to tertiary care. The mean of hospital‐level means for enrollees at these 44 High Rural Serving hospitals was 40,270 (range: 9954–71,792).

## 4 Discussion

Although there is a precedent for categorizing healthcare facilities as either rural or urban using geographic location alone [29], this approach risks overlooking other important factors for understanding facility rurality. Geography‐based definitions, for example, may conceal how facilities function (e.g., complexity of services offered, staffing levels) and the unique characteristics of the populations they serve (e.g., the proportion who live rurally) [15, 16]. Since rurality exists along a continuum [30], conceptualizations that move beyond geolocation alone and acknowledge the variety of factors associated with rural residents’ access to care, such as drive times to access services and the complexity of services offered, are necessary and important. Using cluster analysis of 110 VHA acute care hospitals that incorporated nine variables reflecting various aspects of rurality (e.g., hospital complexity, percentage of rural patients, and mean drive times), we identified three distinct clusters of hospitals (i.e., Low, Medium, and High Rural Serving) that reflect the services provided and populations served.

The average proportion of rural and highly rural veterans and mean drive times differed between the three clusters. Across the clusters, mean drive times to primary, secondary, and tertiary care increased incrementally, with the largest drive times found among hospitals in the High Rural Serving cluster (primary care: 29.3 min; secondary care: 62.4 min; tertiary care: 166 min) as compared to those in the Low Rural Serving (primary care: 17.4 min; secondary care: 34.9 min; tertiary care: 63.7 min) and Medium Rural Serving (primary care: 21.5 min; secondary care: 49.1 min; tertiary care: 68.9 min) clusters. In the High Rural Serving cluster, where nearly two‐thirds of enrollees were rural, hospitals also comprised a mixture of low, medium, and high complexity facilities. Interestingly, the mean drive times found in the High Rural Serving cluster are similar to VHA drive time standards outlined in the MISSION Act for primary care (30 min) and specialty care (60 min) that determine eligibility for community care paid for by VHA [31].

While hospitals in the Low Rural Serving cluster were largely high complexity, hospitals in the High Rural Serving cluster were a mixture of low, medium, and high complexity. A low complexity hospital in the High Rural Serving cluster, where veterans must travel on average over 160 min to access tertiary care, may experience different challenges around care coordination and patient transportation than a high complexity hospital in the Low Rural Serving cluster. While not assessed by this study, findings may inform future work aimed at identifying hospitals’ unique needs for support based on their cluster designation.

The hospital classifications identified in this work may have important implications for diverse audiences including healthcare leaders, policy makers, and researchers. For healthcare leaders, being able to classify a hospital as more or less rural can impact the types of services provided and their understanding of the population served. For example, knowing that a hospital serves a disproportionate number of rural patients may encourage hospital leadership to support implementing healthcare improvement initiatives that address rural patients’ access barriers and offering targeted outreach to rural patients to inform them of their care options. Initiatives may include expanding transportation options for rural patients, enhancing the availability of services offered via telehealth or hybrid models of care, training providers in using telehealth effectively, and providing rural patients with digital literacy training and/or personalized support to facilitate their use of remotely delivered care. Importantly, focusing on a hospital's geographic location alone rather than the patients it serves, particularly for hospitals located in urban locations, may result in missed opportunities for hospital leaders to implement rural‐focused programming.

Policy makers can use rural hospital classifications to allocate funding or other resources based on the needs of the population, whether rural or urban, rather than simply relying on geolocation. Given rising healthcare costs and pressures to maximize efficient allocation of resources, relevant and appropriate designations around rurality can aid in ensuring that resources go where they are most needed. For example, policy makers may consider expanding eligibility for rural‐focused programs like transportation reimbursement or van services to include hospitals that, despite their urban location, serve disproportionately large numbers of rural residents. Doing so would acknowledge the reality that many rural residents must travel to urban areas to receive hospital care not available in rural areas. Future versions of this classification system could also be used to inform reimbursement models like Medicare cost plus reimbursement used for Critical Access Hospitals. In other words, sites in the High Rural Serving cluster could hypothetically qualify for enhanced reimbursement for healthcare services, similar to how Medicare supports low‐volume or resource‐limited facilities through cost‐based (cost plus) reimbursement. In this hypothetical scenario, sites would receive enhanced reimbursement in recognition of the fact that lower volume care often results in narrower profit margins and thus less financial stability for clinics. Changes to eligibility criteria must be made thoughtfully and carefully, however, given that expanding definitions too broadly risks funneling resources away from rural facilities and the communities who need them [32].

Given that many healthcare improvements and innovations are driven by research [33], these classifications may be useful to researchers inside and outside the VHA as well as those who fund rural‐related programs and projects. Knowing which hospitals serve large numbers of rural patients allows researchers to more effectively estimate the potential impact of their projects and can enable more targeted recruitment efforts based on where rural patients receive their care. For example, researchers interested in recruiting rural veterans receiving emergency care could use these clusters to identify acute care hospitals serving large numbers of rural veterans. This three‐tiered clustering provides another option beyond a dichotomous urban/rural comparison and is based upon the population served, drive times, and complexity of services provided. Similarly, those who fund rural health research can use these classifications to consider which proposed projects are more likely to successfully engage rural patients as participants. Funders reviewing a proposal that aims to recruit rural veterans may use these classifications to better assess whether the proposed study is likely to engage, enroll, and thus benefit rural veterans. In healthcare systems like the VHA, having a more nuanced conceptualization of rural hospitals is essential for demonstrating that research projects can improve patients’ access to care and healthcare experiences.

Although our findings were derived from VHA data, they have implications that extend beyond the VHA. Most non‐VHA hospitals that primarily serve rural residents are either Critical Access Hospitals or Rural Emergency Hospitals [34]. These hospitals are classified by the Center for Medicare and Medicaid Services (CMS) to determine reimbursement and types of services offered [35]. CMS's classification system uses a combination of factors to determine these two designations including hospital location, number of inpatient beds, average length of stay, provision of emergency care, and distance to another hospital [36, 37]. Although geolocation can be used to assign rurality to the physical location of the hospital, this provides limited discrimination between hospitals since most are in urban census tracts, regardless of the rural–urban classification used. Introducing approaches to conceptualizing rural facilities that account for a variety of factors encourages ongoing dialogue and innovation across healthcare systems.

This study has important strengths. The intersectional lens we employed to characterize VHA hospitals is a key innovation of this study. Prior research has primarily used single variables, such as geographic rurality or level of complexity, to study healthcare access and outcomes at VHA hospitals. By taking an intersectional approach, this study demonstrates that such single‐variable approaches mask important hospital heterogeneity. For example, the rurality of the patient populations varied substantially across high‐complexity facilities, which were represented in each identified cluster. Furthermore, this study uncovers that certain VHA hospitals may be providing care in the context of compounded vulnerabilities (e.g., low complexity, lower patient volume, rurally located), which may go unrecognized with single‐variable approaches. For example, several hospitals in the High Rural Serving cluster were low complexity facilities with relatively lower volumes of patient care. Higher volume hospitals may be more likely to provide specialized services and adhere to evidence‐based clinical guidelines than lower volume hospitals [38]. The rurality of hospitals’ patient populations and the services they do and do not provide may interact to shape health outcomes in unique ways that require tailored policy and programmatic solutions.

There are also limitations of this work to consider. First, the analysis is limited to only nine variables. Future analyses should explore additional variables meaningful for understanding hospital‐level rurality. For example, we did not include hospital staffing levels, hospital proximity to an academic medical center, proximity to non‐VHA community facilities or providers, or hospital financial data, which may have important implications for rural veterans’ access to care. The current study thus represents an initial step in characterizing rural VHA hospitals beyond geography‐based classification alone; additional work is needed to refine and validate the model prior to wide‐scale implementation. Second, as an analysis, k‐means clustering is sensitive to outliers, and different initializations can lead to different results (i.e., selection of number of k initial centroids). We mitigated this risk of bias by using 50 random initializations to improve solution stability. However, additional initializations may have impacted study results. Third, there are limitations to the VHA data included in this work. PSSG 2023 enrollee file data do not include veterans who have enrolled in VHA after FY2023 and calculated average drive times may not factor in considerations like real‐world road and driving conditions. Finally, these analyses were limited to veterans enrolled in VHA and the 110 acute care hospitals where they can receive VHA services. It may not directly apply to other healthcare systems or non‐VHA sites of care. Future directions involve expanding the analyses to include additional variables which may reflect aspects underlying rurality and types of VHA facilities (i.e., community‐based outpatient clinics) and non‐VHA providers in VHA's Community Care Network.

In conclusion, this work provides a novel approach for discriminating hospitals into three categories of Low Rural Serving, Medium Rural Serving, and High Rural Serving based upon the population served, the complexity of the healthcare services offered at each facility, and drive times to care facilities. These categories can provide valuable guidance for a variety of VHA and non‐VHA professionals working to enhance rural residents’ access to care. Future work should test the predictive validity of the clusters, expand the model to include other factors relevant to characterizing rural facilities, and further develop analyses to include other VHA and non‐VHA facilities and services.

## Funding

This material is based upon work supported (or supported in part) by the US Department of Veterans Affairs (VA), Veterans Health Administration (VHA), Office of Rural Health (ORH), Veterans Rural Health Resource Center‐Iowa City Rural Health Career Development Awards (#04269) to Alexandra Caloudas and Diana Govier, VHA Office of Research and Development, Health Systems Research Service (HSR) through the Comprehensive Access and Delivery Research and Evaluation Center (CIN 13‐412), HSR Center for Innovations in Quality, Effectiveness and Safety (CIN 13‐413), and South Central VA Health Care Network, South Central Mental Illness Research, Education and Clinical Center. Drs. Lovejoy and Ono were supported by the VHA Office of Rural Health (PROG‐0000095) during the conduct of this study.

## Conflicts of Interest

This manuscript is not under review elsewhere and there is no prior publication of manuscript contents. The views expressed in this article are those of the authors and do not necessarily represent the views of the US Department of Veterans Affairs, the United States Government, or any other entities with whom the authors are affiliated. The authors report no conflict of interest regarding this work.
